# Current News and Opinion

**Published:** 1882-09

**Authors:** 


### CURRENT NEWS AND OPINION.

DR. A. S. HEATON, Professor of Clinical Medicine in Detroit College, died in that city on July 9, in the fifty-fourth year of his age.

DR. J. MARION SIMS has been again honored abroad. The French Government has just created him an officer of the Legion of Honor, in recognition of his services in the organization of the ambulance service during the Franco-Prussian war.

FOSTER'S Physiology has been translated into Italian by M. Lessona, of Milan.

DR. ZINSSER, of New York, reports (*N. Y. Medical Journal*, Sept.) a case of bilateral herpes zoster. This form of the disease is quite rare, and the popular belief that it is invariably fatal when it crosses the middle line of the body is still widely diffused.

DURING the month of July the total number of passengers arriving in New York from Europe was 18,513.

SIX medical journals are published in Japan in the native language.

THE AMERICAN Dermatological Association held its sixth annual meeting in Newport August 30 to September 1, inclusive. A considerable number of valuable papers were read. The officers elected for the ensuing year were: Dr. R. W. Taylor, *President;* Drs. J. E. Atkinson and A. R. Robinson, *Vice-Presidents;* Dr. A. Van Harlingen, *Secretary*, and Dr. George H. Rohé, *Treasurer.* The next meeting will be held at Lake George.

THE ROYAL College of Physicians has passed a resolution declaring that "the custom of giving laudatory certificates of medicinal and other preparations and medical and surgical appliances, whether for publication or not, is misleading to the public, derogatory to the dignity of the profession, and contrary to the traditions and resolutions of the Royal College of Physicians."

To read the advertisements of various mineral waters in the daily papers, one would be led to think the "traditions and rules of the Royal College of Physicians" had not been very closely followed of late.

The Argentine prize of 10,000 francs has been divided between Dr. H. J. Bigelow and Dr. Th. Anger for advances in genito-urinary surgery. Dr. Bigelow receives 6,000 francs and Dr. Anger the balance.

A cheap disinfectant is said to be the following: Dissolve half a dram of nitrate of lead in a pint of boiling water; also two drams of common salt in a bucket of water; pour the two solutions together; allow the sediment to precipitate, and the clear, supernatant fluid will be a chloride of lead. This will deodorize and disinfect sinks, water-closets or drains, and a cloth dipped into it will sweeten a fetid atmosphere.

The popularity of dentistry is one of the mysteries of professional life. Why should so many young men, who might easily become physicians or surgeons, restrict their energies to the human jaw instead of taking in the whole body? We hope the explanation is not to be found in an episode of this morning's police news, in which one of these young men figures as embracing along with his profession a fair patient in the chair. If there are social reasons for this professional preference it may be interesting to ascertain whether there are dentists in countries where kissing is not in vogue. The Yakuts along the Lena, for example, do not favor this form of embrance, and as a matter of fact there is not a dentist to be found in their villages. We have supposed that the absence of this useful member of society could be explained by the presence of the finest teeth in the world in the mouths of the natives. But this may be a superficial view.

A Student of Women.—A French physician advocates some novel theories regarding women, and claims to know about them as well as anyone can who has dissected them much and loved them much!

